# Distance and density dependence in two native Bornean dipterocarp species

**DOI:** 10.1002/ece3.10004

**Published:** 2023-04-19

**Authors:** Nazrin Malik, David Edwards, Robert P. Freckleton

**Affiliations:** ^1^ Ecology and Evolutionary Biology, School of Biosciences University of Sheffield Sheffield S10 2TN UK; ^2^ Department of Forestry Science & Biodiversity, Faculty of Forestry Universiti Putra Malaysia Serdang Selangor 43400 Malaysia

**Keywords:** coexistence, density dependence, dipterocarpaceae, distance dependence, diversity, Janzen–Connell hypothesis, seedling, survival

## Abstract

The Janzen–Connell hypothesis proposes that density and distance‐dependent mortality generated by specialist natural enemies prevent competitive dominance. Much literature on Janzen–Connell mechanisms comes from the neotropics, and evidence of the role of distance and density‐dependence is still relatively sparse. We tested the predictions of the Janzen–Connell hypothesis in a South‐East Asian system dominated by mast fruiting species. We hypothesized that seedling survival would decrease with distance and density, seedling growth would increase, and herbivory would decrease, according to the predictions of the Janzen–Connell hypothesis. Experiments were conducted to determine the strength of the Janzen–Connell mechanism by manipulating the density and identity of tree species as a function of the distance from parent trees. Survival of conspecific seedlings was reduced near adult trees of one species, but not another. High densities of seedlings decreased the growth of conspecific seedlings of both species. In both species, herbivory rates decreased with distance in low‐density areas. This study indicates that dipterocarp species experienced weak Janzen–Connell effects of distance and density dependence at the growth stage studied. Future studies in this system might focus on earlier life‐history stages such as seeds and small seedlings, as well as studying mortality during mast‐seeding events.

## 1 INTRODUCTION

Tropical rainforests are the most diverse ecosystems in terms of community structure and species diversity (Chazdon, 2003; Edwards et al., 2014; Gardner et al., 2009; Steege et al., 2015). It has been a challenge for ecologists to understand the process that maintains diversity in plant communities, and this is especially true in hyperdiverse tropical forests (Bagchi et al., 2014; Chesson, 2000; Dalling et al., 1998; Steege et al., 2015; Terborgh, 2012). In tropical forests the number of species appears to greatly exceed the number of limiting resources (Hutchinson, 1961). Under such circumstances the competitive exclusion principle predicts that the superior species will drive other species to extinction (Hardin, 1960; Levin, 1970).

Various hypotheses have been proposed to explain the diversity of tropical forests (Connell, 1971; Connell, 1978; Hubbell, 2001; Janzen, 1970; Schoener, 1974). Niche partitioning is a mechanism that explains high diversity through minimizing competition between species (Schoener, 1974). Kraft et al. (2008) showed that niche partitioning can contribute to the maintenance of forest diversity, and other studies have indicated that there is niche partitioning in dipterocarp forests (Gunatilleke et al., 2006; Potts et al., 2002). However, this mechanism is unlikely on its own to explain coexistence (Barot, 2004; Brown et al., 2013; Wright, 2002).

One of the leading theories for explaining tropical forest diversity is the Janzen–Connell hypothesis (Connell, 1971; Janzen, 1970). The Janzen–Connell hypothesis suggests that specialized natural enemies (pathogens, seed predators, and herbivores) play a vital role in maintaining the diversity of tropical plant species in a density‐dependent manner. This works through reducing the survival of seeds and seedlings near conspecific adults where seed density is the highest. According to this hypothesis, if natural enemies are sufficiently specialized, they aggregate on high densities of seeds or seedlings of their hosts close to adult trees (Dalling et al., 1998; Freckleton & Lewis, 2006; Fukue et al., 2007; Hülsmann et al., 2021; Swamy & Terborgh, 2010; Traveset, 1990). The density‐dependent nature of the resultant mortality prevents competitive exclusion (Comita & Stump, 2020). This is because locally abundant species will experience higher mortality than rare ones, thus allowing the rarer species to survive and coexist. This density‐dependence acts as a stabilizing mechanism that can promote the maintenance of diversity (Chesson, 2000).

Based on field experiments there is growing evidence that natural enemies play a role in generating density and distance‐dependent mortality (Bagchi, Press, & Scholes, 2010; Bagchi, Swinfield, et al., 2010; Brook & Bradshaw, 2006; Comita et al., 2014; Comita & Stump, 2020; Song et al., 2021; Song & Corlett, 2021). There is substantial evidence that seed survival increases with distance from the conspecific adults while high densities of seed or seedlings increase mortality in the tropics (Massey et al., 2006; Matthesius et al., 2011; Norghauer et al., 2006; Peres & Baider, 1997; Swamy & Terborgh, 2010; Terborgh et al., 1993), as well as some evidence in temperate systems as well (Jia et al., 2020; Packer & Clay, 2000).

Despite an accumulation of evidence, there are some limitations and gaps in the literature, however. Studies on Janzen–Connell effects come from the Neotropics, particularly in Central and South America (Augspurger & Kitajima, 1992; Dalling et al., 1998; Forget, 1992; Peres & Baider, 1997; Roberts & Heithaus, 1986; Sanchez‐cordero & Martinez‐gallardo, 2010; Stevenson et al., 2005; Swamy & Terborgh, 2010). By comparison, there is a relative dearth in Africa (Chapman & Chapman, 1996; Hart, 1995; Matthesius et al., 2011), and Asia (Bagchi, Press, & Scholes, 2010; Bagchi, Swinfield, et al., 2010; Krishnan et al., 2022; Massey et al., 2006; Takeuchi & Nakashizuka, 2007; Viswanathan et al., 2020).

This geographic gap is important to address because, globally, forests differ from each other in important ways. For example, Asian dipterocarps are unique for their mast reproduction and fruiting. In Southeast Asian forests, the dominant Dipterocarp species are usually involved in community wide mast fruiting events (Appanah, 1993; Ashton, 1988). It has been hypothesized that systems undergoing mast‐fruiting may not experience strong density and distance‐dependent predation because of predator satiation (Cannon et al., 2021; Curran & Webb, 2000; Webb & Peart, 1999). This is because all species produce large numbers of seeds simultaneously, and there will be insufficient predators to generate significant mortality.

Several studies have found that predator satiation, especially in Dipterocarps, weakens the Janzen–Connell mechanism (Ashton, 1988; Curran & Webb, 2000; Paoli et al., 2006). Several characteristics of Dipterocarp seeds and seedlings such as large size, poor chemical defense, and being energy rich make them attractive food for wild pigs, *Sus barbatus* (Ashton, 1988; Curran & Webb, 2000), and weevil beetles, family: Curculionidae (Bagchi et al., 2011; Lyal & Curran, 2000). From the perspective of maintaining diversity, generalist natural enemies are expected to have a low diversity‐enhancing effect compared with specialists (Curran & Leighton, 2000; Freckleton & Lewis, 2006; Gilbert, 2005). Theory suggests that generalist natural enemies should not generate Janzen–Connell mechanisms (Freckleton & Lewis, 2006). More recent work has shown that limited amounts of generalism can nevertheless still yield diversity enhancement (Sedio & Ostling, 2013). Bagchi, Swinfield, et al. (2010); Bagchi, Press, and Scholes (2010) have shown evidence for distance‐dependence in dipterocarps, however overall, there is little understanding of the role of Janzen–Connell mechanisms in hyperdiverse forests with mast‐seeding.

Here we examine the effect of distance and density on two Bornean dipterocarp species, *Parashorea malaanonan* and *Shorea johorensis*. We manipulated the density and type of tree species (*Parashorea malaanonan* and *Shorea johorensis*) as a function of the distance from conspecific adult trees. We experimentally tested the strength of Janzen–Connell hypothesis in these two native dipterocarp species, specifically addressing the following hypotheses: (1) the survival of conspecific seedlings will decrease with proximity to conspecific adult trees (distance‐dependence) and within high density of conspecific seedlings (density‐dependence) compared to heterospecific seedlings; (2) high density of conspecific seedlings will decrease the growth of conspecific seedlings; (3) Herbivory rates in conspecific seedling will decrease with increasing distance from conspecific adult trees; and (4) Leaf herbivory in new leaves decrease with increasing distance from conspecific adult trees.

## 2 MATERIALS AND METHODS

### 2.1 Study system

This study was conducted at the Danum Valley Field Centre, Sabah, East Malaysia (4° 58′ N, 117°48′ E) which is located at eastern border of Danum Valley Conservation Area (DVCA). Danum Valley Conservation Area (Class 1 forest reserve) is 43,800 ha of primary lowland dipterocarp forest with relatively little human disturbance (Marsh & Greer, 1992). The soils in DVCA are orthic acrisols, developed on sandstone and mudstone. Clay percentage in these soils ranging from 30% to 60% with acidity ranges from 5.3 to 4.0. The mean minimum and maximum temperature at the field center is 22.6 and 31.2°C respectively, while mean annual rainfall is 2881 mm (Walsh et al., 2011).

The Dipterocarpaceae is a family of hardwood trees, and is typically the dominant family in the tropical forests of South East Asia (Ashton, 1988). Although this family is generally found in South East Asia, India, Sri Lanka, Philippines, Madagascar, Africa and Papua New Guinea (Ådjers et al., 1995; Appanah, 1993; Ashton, 1988), Borneo is the region with highest diversity of Dipterocarpaceae (Ashton, 1982). Dipterocarps fruits are large and winged but usually dispersed over shorter distances (≤60–80 m) in closed‐canopy forest (Smits, 1994; Whitmore, 1984). Dipterocarpaceae generally exhibit community‐wide mast fruiting events (Ashton, 1988; Curran & Leighton, 2000), typically during El Nino years (Ashton, 1988; Bebber et al., 2004).

Our study focuses on two dominant dipterocarps in the region, *Parashorea malaanonan* and *Shorea johorensis. Parashorea malaanonan* is one of the native dipterocarp species in this region (18.6 stems/ha) (Stoll & Newbery, 2005). *Parashorea malaanonan* is classified as White Seraya Light Hardwood and known as a fast‐growing dipterocarp species in Borneo (Bagchi, Press, & Scholes, 2010; Bagchi, Swinfield, et al., 2010). *Shorea johorensis* is native dipterocarp species, fast‐growing and big emergent trees that can usually be found in Danum Valley Conservation Area, with 24.6 stems/ha (Brown & Whitmore, 1992; Stoll & Newbery, 2005). It belongs to Light Red Meranti group, and frequently used in plywood and veneer. Following recent community‐wide mast fruiting events, seedlings of these two dipterocarp species were easy to locate and are often intermingled.

### 2.2 Field experiment

Conspecific adult trees of *P. malaanonan* and *S. johorensis* were located by searching along a 2 km network of trails adjacent to the field center. These two species were distinguished in the field based on their key characteristics (Soepadmo et al., 2004).

At each conspecific adult tree (diameter at breast height > 30 cm), one transect was set up from 2 m to 30 m away from conspecific adult tree. We checked that there were no adult trees within a distance of 30 m of each parent. Twelve 1 m × 1 m plots (1 m^2^) were established along each transect, consisting of four experimental plots each at distances 2 m, 15 m, and 30 m from the conspecific adult tree, respectively (following Bagchi, Swinfield, et al., 2010; Bagchi, Press, & Scholes, 2010). Each plot was randomly assigned to one of four treatments: (1) low density of seedlings (4 seedlings m^−2^), (2) high density of seedlings (12 seedlings m^−2^), (3) mixed species with low density of seedlings, and (4) mixed species with high density of seedlings. The above setup was replicated for 10 trees of each species (i.e., 240 quadrats were established in total for both species).

Seedlings of the two species were obtained from the Innoprise—FACE Foundation Rainforest Rehabilitation Project (INFAPRO) nursery, near Danum Valley Field Centre. Currently, this nursery has stocks of 28 native dipterocarps species and six other indigenous species. All the dipterocarp seedlings in this nursery are collected from recent mast fruiting events. Germinated seeds of the two‐study species were planted in polybags in July 2014 and kept in the nursery: thus, the seedlings used in this study were 2 years old.

In all experimental plots, existing plants were removed but leaf litter on the ground was left. Seedlings were planted using a planting bar. This is used to prepare holes for seedlings planting. Planting bars provide suitable holes for small seedlings particularly in small plots and prevent excessive disturbance to the forest soil. In total across all treatments 96 seedlings were planted in 12 plots (four plots for each distance) adjacent to each conspecific adult tree.

### 2.3 Measurements

All seedlings were tagged with numbered aluminum labels and identified to species (or to the lowest taxonomic level possible) with the help of a botanist. The heights of all seedlings were measured by using a 1 m rule. Seedlings height were measured at the beginning of the experiment and at the end of the experiment. Stem diameters were measured just below the cotyledon scar using a digital vernier caliper (Haase, 2008). All leaves surviving from the first census and new leaves produced during the interval were recorded for each seedling.

In order to estimate the rate of herbivory, five leaves were selected from each seedling and labeled with a unique number written in permanent ink on the underside of leaves during the first census (July 2016). Visual estimates were employed in this study where herbivory damage is estimated as the percentage of leaf surface area removed (Stotz et al., 2000). All seedlings were re‐measured in June 2017. The number of marked leaves missing, and herbivory of new leaves also were recorded. In each plot, a spherical densiometer was used to determine canopy openness and light availability to seedlings (Lemmon, 1956).

### 2.4 Statistical analyses

The survival and growth data were analyzed separately for each of the focal species. To test for effects of distance and density treatment on survival of conspecific seedling, seedling data were analyzed using generalized linear models (GLMs) with a quasi‐binomial distribution and logit link function (Survival ~ as.factor (Tree) + Distance * Species Identity * Density). The quasi‐binomial distribution was used to account for overdispersion. To analyze the effects of distance and density on growth and herbivory of planted seedlings, a linear model was used (Height/Diameter/Herbivory ~ as.factor (Tree) + Distance * Density * Mixture. monoculture). All statistical analyses were conducted in the statistical software environment R version 4.2.0 (R Core Team, 2022).

## 3 RESULTS

### 3.1 Effects of distance and density on survival of seedlings

**FIGURE 1:**
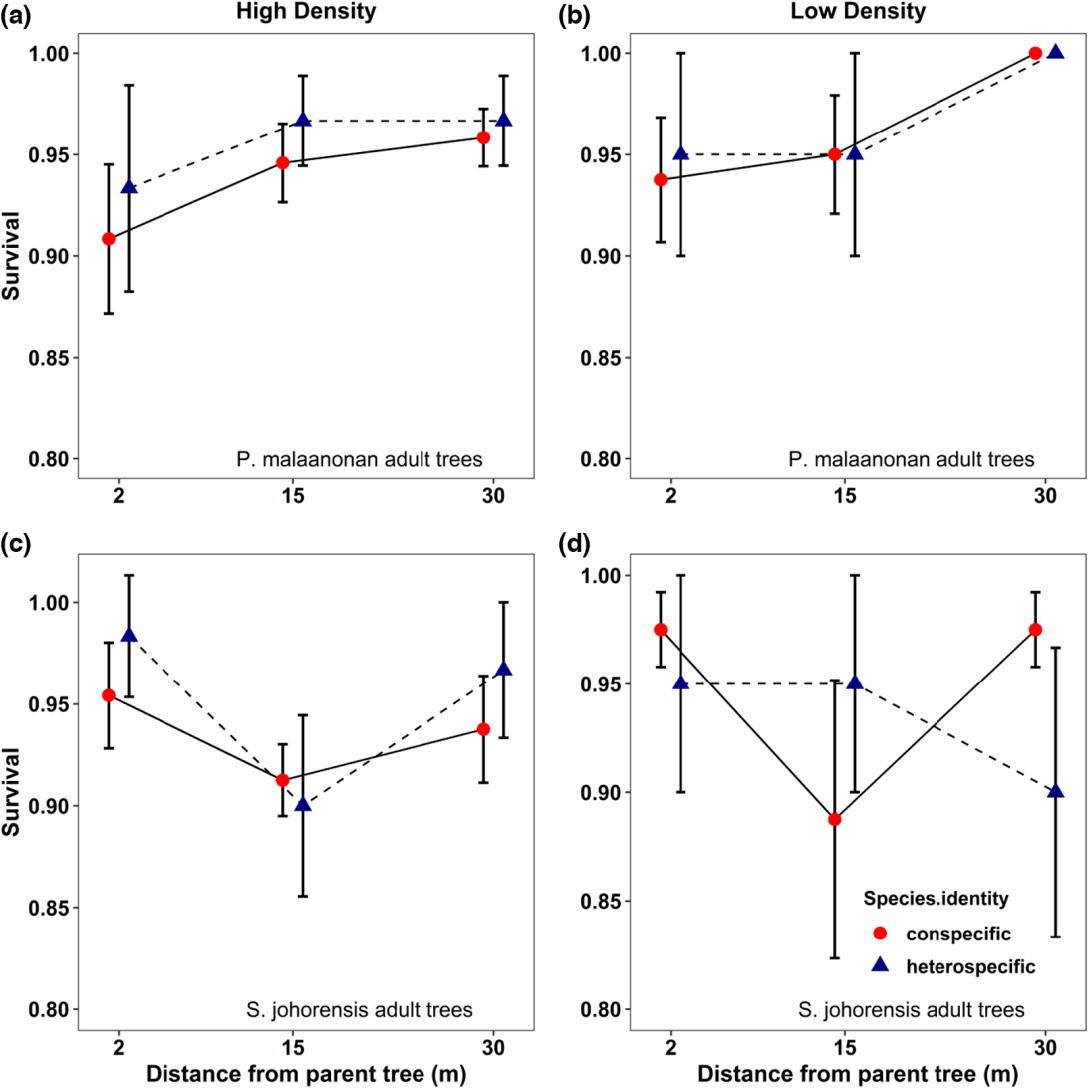
Seedling survival at conspecific and heterospecific seedlings as a function of distance from *P. malaanonan* adult trees at high (a) and low (b) and distance from *S. johorensis* adult trees at high (c) and low (d) in distance‐density experiment. Error bars represent standard error of the mean after transforming to the proportion scale.

There was a significant effect of distance from *P. malaanonan* adult trees on the survival of seedlings (Table 1; *F*_1, 169_ = 9.544, *p* = .002). Survival of conspecific and heterospecific seedlings was highest at the furthest distance (30 m) while lowest at the nearest distance (2 m) (Figure 1a). Closest to the adult (2 m), conspecific seedlings suffer higher mortality compared to heterospecific seedlings in both high‐ and low‐density treatment. However, there was no marginal effect of density on survival when the distance to adults was statistically controlled (Table 1; *F*_1, 167_ = 2.279, *p* = .133).

**TABLE 1:** Summary of analysis of variance tables for generalized linear model to investigate the effect of distance and density treatment on survival of seedlings around *Parashorea malaanonan* and *Shorea johorensis* adult trees. Terms were tested sequentially.

	*Parashorea malaanonan*			*Shorea johorensis*		
	df	Resid df	*F* (*p*‐value)	df	Resid df	*F* (*p*‐value)
Tree	9	170	4.691 (1.518e−05))***	9	170	0.899 (.528)
Distance	1	169	9.544 (.002)**	1	169	0.389 (.534)
Species identity	1	168	1.254 (.264)	1	168	0.027 (.869)
Density	1	167	2.279 (.133)	1	167	0.019 (.891)
Distance * Species identity	1	166	0.006 (.939)	1	166	0.001 (.972)
Distance * Density	1	165	1.585 (.210)	1	165	0.005 (.944)
Species. Identity * Density	1	164	0.089 (.766)	1	164	0.170 (.681)

*Note*: Values in the bracket is *p*‐value.

*Significant level at *p* < .05.

** Significant level at *p* < .01.

*** Significant level at *p* < .01.

Around *S. johorensis* adult trees, there was no significant trend in survival with distance for seedlings of both species (Table 1; *F*_1, 169_ = 0.389, *p* = .534). Furthermore, no significant effects of density on conspecific or heterospecific seedlings survival (Table 1; *F*_1, 167_ = 0.019, *p* = .891).

### 3.2 Effects of distance and density on growth of seedlings

**FIGURE 2:**
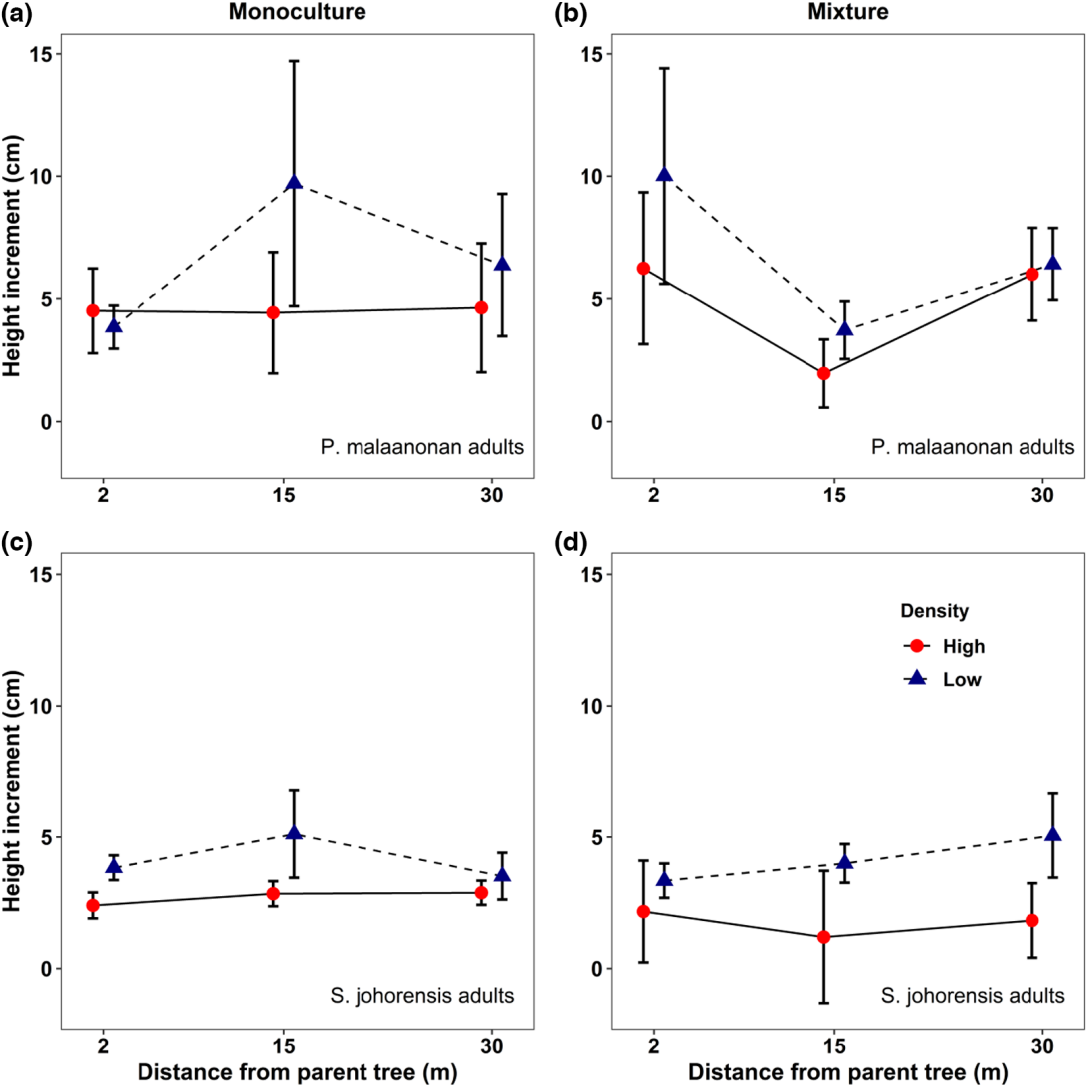
Effects of distance and density on height increment of conspecific seedlings from adult *P. malaanonan* (a, b) and *S. johorensis* (c, d) trees with monoculture (a, c) or mixture (b, d) planting treatment. Error bars represent standard error of the mean.

Overall, there was evidence that density affected the growth of seedlings, however little evidence of an effect of distance. There was a significant effect of density on height increments of both conspecific seedlings of *P. malaanonan* (Figure 2a,b, Table 2; *F*_1, 96_ = 4.679, *p* = .033) and *S. johorensis* (Figure 2c,d, Table 2; *F*_1, 96_ = 4.970, *p* = .028). Height increment conspecific seedlings of *P. malaanonan* increased by 5.27 cm in low‐density plot compared to high‐density plot. For *S. johorensis* conspecific seedlings, height increment increased by 2.27 cm in low‐density plot compared to high‐density plot. However, no significant effect of distance from adult tree was observed on height increment for both conspecific seedlings for *P. malaanonan* (Table 2; *F*_1, 96_ = 0.031, *p* = .860) and *S. johorensis* (Table 2; *F*_1, 96_ = 0.904, *p* = .344).

**TABLE 2:** Summary of analysis of variance tables of linear models on effect of distance and density on growth increment for conspecific seedlings at *Parashorea malaanonan* and *Shorea johorensis* adult trees. Terms were tested sequentially.

	*Parashorea malaanonan*		*Shorea johorensis*	
	df	*F* (*p*‐value)	df	*F* (*p*‐value)
*Log height*				
Tree	9	2.831 (.005)**	9	2.916 (.004)**
Distance	1	0.031 (.860)	1	0.904 (.344)
Density	1	4.679 (.033)*	1	4.970 (.028)*
Mix.mono	1	0.188 (.665)	1	0.685 (.409)
Distance * Density	1	0.156 (.694)	1	0.334 (.565)
Distance * Mixed/Monoculture	1	0.025 (.875)	1	0.690 (.408)
Density * Mixed/Monoculture	1	0.220 (.640)	1	0.525 (.471)
*Log diameter*				
Tree	9	2.833 (.005)**	9	1.976 (.049)*
Distance	1	0.145 (.705)	1	7.013 (.009)**
Density	1	1.519 (.221)	1	10.724 (.001)**
Mixed/Monoculture	1	5.438 (.022)*	1	1.688 (.197)
Distance * Density	1	0.163 (.688)	1	4.304 (.041)*
Distance * Mixed/Monoculture	1	0.559 (.456)	1	0.216 (.643)
Density * Mixed/Monoculture	1	3.988 (.048)*	1	0.159 (.691)
*Log number of leaves*				
Tree	9	1.563 (.136)	9	8.429 (2.542e‐09)
Distance	1	0.791 (.376)	1	0.050 (.824)
Density	1	0.271 (.604)	1	1.354 (.247)
Mixed/Monoculture	1	2.547 (.114)	1	0.259 (.611)
Distance * Density	1	0.005 (.947)	1	0.035 (.852)
Distance * Mixed/Monoculture	1	0.023 (.880)	1	3.172 (.078)
Density * Mixed/Monoculture	1	1.501 (.223)	1	0.843 (.361)

*Note*: Values in the bracket is *p*‐value.

* Significant level at *p* < .05.

** Significant level at *p* < .01.

**FIGURE 3:**
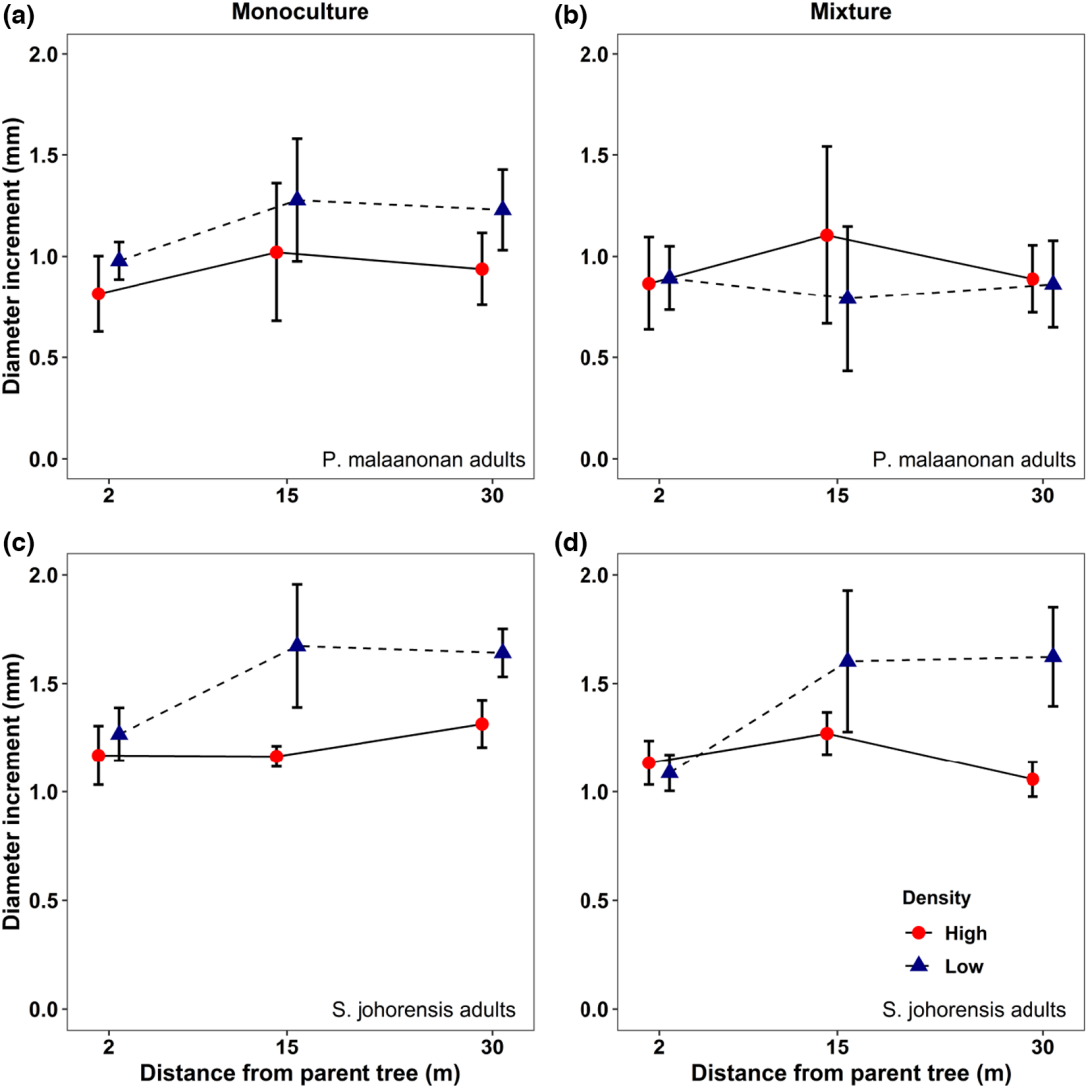
Effects of distance and density on diameter increment of conspecific seedlings from adult *P. malaanonan* (a, b) and *S. johorensis* (c, d) trees with monoculture (a, c) or mixture (b, d) planting treatment. Error bars represent standard error of the mean.

There was a significant effect of mixture and monoculture planting treatment on diameter increment of conspecific seedlings around trees of *P. malaanonan* (Figure 3a,b, Table 2; *F*_1, 103_ = 5.438, *p* = .022). Diameter increment of conspecific seedlings around trees of *P. malaanonan* increased by 0.6 cm in monoculture plot compared to mixed species planting treatment plot. A weakly significant interaction was observed between the density treatment and the mixed and monoculture planting treatments (Table 2; *F*_1, 103_ = 3.988, *p* = .048).

Surrounding *S. johorensis* adult trees, there was a significant positive effect of distance on diameter increment of conspecific seedlings (Figure 3c,d: *F*_1, 102_ = 7.013, *p* = .009). Diameter decreased by 0.56 cm between near and far treatments. Furthermore, a significant positive effect of density treatment was also observed on diameter increment of conspecific seedlings (*F*_1, 102_ = 10.724, *p* = .001). Diameter increment conspecific seedlings of *S. johorensis* increased by 0.51 cm in low‐density plot compared to high‐density plot. There was a significant interaction between distance and density (*F*_1, 102_ = 4.304, *p* = .041). Thus, diameter increment increased with distance from adult trees at low, but not high densities (see Table 2).

We found no significant effect of distance and density treatment on number of leaves of conspecific seedlings around both *P. malaanonan* and *S. johorensis* adult trees (Table 2).

### 3.3 Effects of distance and density on herbivory

**FIGURE 4:**
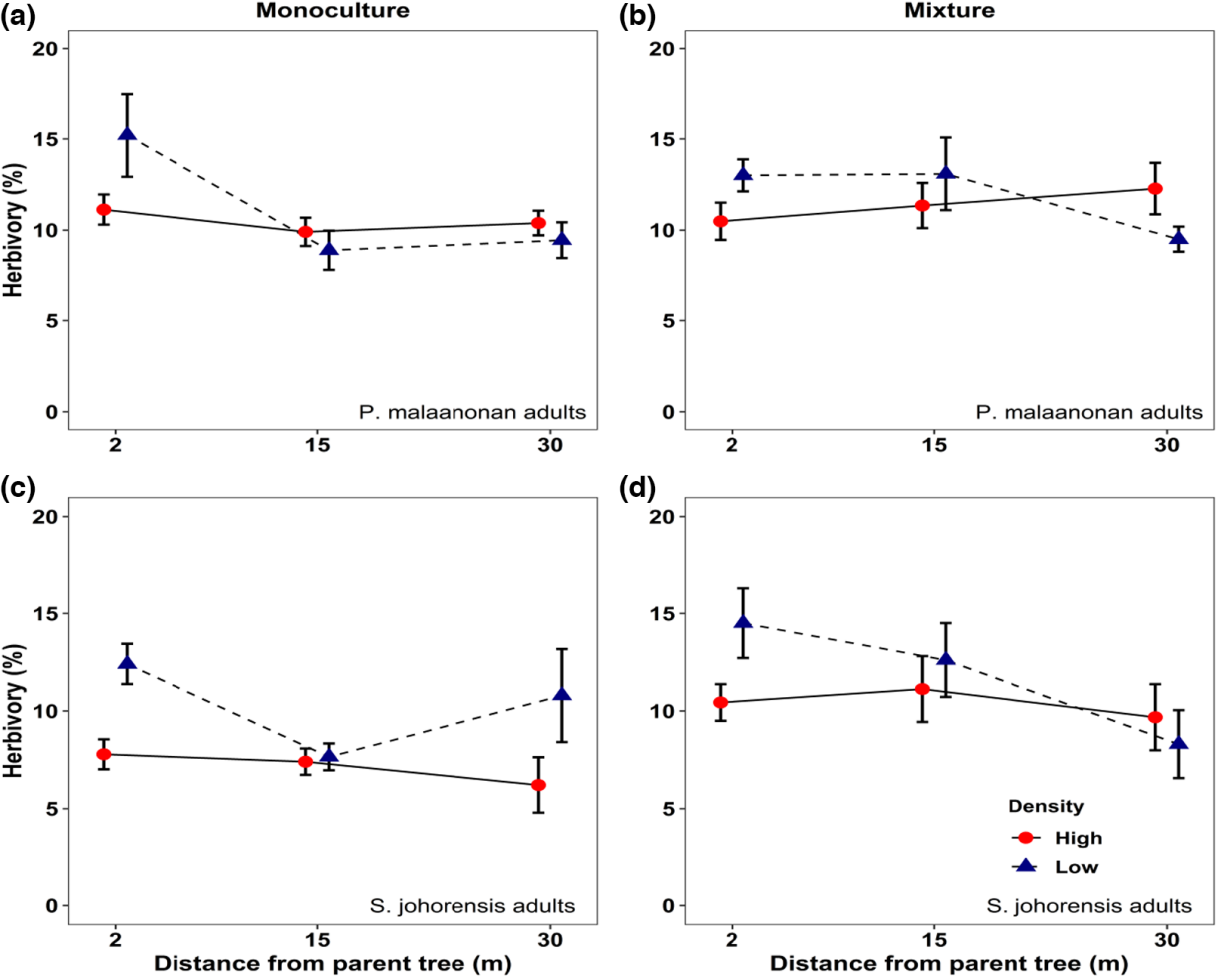
Effects of distance and density on herbivory rates of conspecific seedlings from adult *P. malaanonan* (a, b) and *S. johorensis* (c, d) trees with monoculture (a, c) or mixture (b, d) planting treatment. Error bars represent standard error of the mean.

There was a consistent decline in herbivory with distance from parents of both species, as well as evidence for impacts of density as well (Table 3). In the low‐density treatment, herbivory rates of *P. malaanonan* seedlings and *S. johorensis* decreased with distance from adult *P. malaanonan* trees (Figure 4) (*F*_1, 103_ = 5.675, *p* = .019). A significant interaction was observed between distance and density variables (*F*_1, 103_ = 9.165, *p* = .003), with a negative effect of distance in the low density, but not the high‐density treatment (Figure 4a,b).

Around *S. johorensis* adult trees, there was a significant effect of distance on herbivory rate on *S. johorensis* seedlings indicating that herbivory rates decreased with distance from adult trees (*F*_1, 102_ = 6.363, *p* = .013). The herbivory rate of *S. johorensis* seedlings was negatively affected by seedling density as low density exhibited high herbivory rates compared to high‐density plots (*F*_1, 102_ = 7.969, *p* = .006). Furthermore, mixture and monoculture planting treatment also had a highly significant effect on herbivory rates in *S. johorensis seedlings* (Figure 4c,d, *F*_1, 102_ = 9.038, *p* = .003) (see Table 3).

**TABLE 3:** Summary of analysis of variance tables of linear models on effect of distance and density on herbivory rates of conspecific seedlings at *Parashorea malaanonan* and *Shorea johorensis* adult trees. Terms were tested sequentially.

	*Parashorea malaanonan*		*Shorea johorensis*	
	df	*F* (*p*‐value)	df	*F* (*p*‐value)
Tree	9	2.542 (.011)*	9	2.275 (.023)*
Distance	1	5.675 (.019)*	1	6.363 (.013)*
Density	1	0.759 (.386)	1	7.969 (.006)**
Mixed/Monoculture	1	1.293 (.258)	1	9.038 (.003)**
Distance * Density	1	9.165 (.003)**	1	1.762 (.187)
Distance * Mixed/Monoculture	1	1.759 (.188)	1	1.031 (.312)
Density * Mixed/Monoculture	1	0.025 (.875)	1	1.012 (.317)

*Note*: Values in the bracket is *p*‐value.

* Significant level at *p* < .05.

** Significant level at *p* < .01.

### 3.4 Effects of distance from parents and density on herbivory of new leaves

There was no significant effect of distance and density on production of new leaves in seedlings of either. We also found no significant effects of distance and density on leaf herbivory in *P. malaanonan* seedlings. However, there was a significant positive effect of distance on herbivory of new leaves in *S. johorensis* seedlings (*F*_1, 108_ = 5.990, *p* = .016). Effects of distance on herbivory varies significantly between density treatment. Thus, there was a significant interaction between distance and density (*F*_1, 108_ = 4.547, *p* = .035) (see Table 4).

**TABLE 4:** Summary of analysis of variance tables of linear models on effect of distance and density on leaf recruitment and herbivory damage of conspecific seedlings at *Parashorea malaanonan* and *Shorea johorensis* adult trees. Terms were tested sequentially.

	*Parashorea malaanonan*		*Shorea johorensis*	
	df	*F* (*p*‐value)	df	*F* (*p*‐value)
*Log Number of new leaves*				
Tree	9	1.185 (.314)	9	1.230 (.285)
Distance	1	0.004 (.947)	1	0.345 (.558)
Density	1	1.945 (.166)	1	1.478 (.227)
Mixed/Monoculture	1	1.877 (.174)	1	0.083 (.774)
Distance * Density	1	0.366 (.547)	1	2.633 (.108)
Distance * Mixed/Monoculture	1	0.001 (.976)	1	0.011 (.916)
Density * Mixed/Monoculture	1	0.433 (.512)	1	0.113 (.738)
*Herbivory of new leaves*				
Tree	9	4.713 (3.572e‐05)	9	3.282 (.073)
Distance	1	0.243 (.624)	1	5.990 (.016)*
Density	1	2.177 (.143)	1	1.949 (.166)
Mixed/Monoculture	1	0.379 (.539)	1	0.229 (.633)
Distance * Density	1	0.290 (.591)	1	4.547 (.035)*
Distance * Mixed/Monoculture	1	2.122 (.149)	1	0.031 (.860)
Density * Mixed/Monoculture	1	3.932 (.050)	1	1.334 (.251)

*Note*: Values in the bracket is *p*‐value.

* Significant level at *p* < .05.

## 4 DISCUSSION

Understanding distance and density‐dependence in plant communities is essential for understanding species diversity in tropical forests (Liu et al., 2012; Schupp & Jordano, 2011). Our study revealed that the two species exhibited contrasting effects of distance and density‐dependence, compared with the predictions of the Janzen–Connell hypothesis. We showed that survival of seedlings located near the adult trees was reduced for *P. malaanonan* indicating distance dependence occurs, but density dependence does not. Distance and density dependence were not detected in *S. johorensis* seedlings. However, we found that in both species, high densities of conspecific seedlings decreased the growth of seedlings. Moreover, herbivory rates on conspecific seedlings of both species decreased with distance. In addition, our study demonstrated that leaf herbivory for new leaves varies with the distance from the focal adult tree.

### 4.1 Effect of distance and density on survival

The effect of distance on survival was stronger for conspecific seedlings than heterospecific seedlings around *P. malaanonan* adult trees, while there was no effect of density on conspecific seedlings. For density‐dependence to promote species coexistence, conspecific seedling must be affected more than heterospecifics (Hille Ris Lambers et al., 2002). Our finding is supported by Bagchi, Swinfield, et al. (2010); Bagchi, Press, and Scholes (2010) who found that survival of naturally occurring *P. malaanonan* seedlings suffered greater reductions near conspecific adult trees than heterospecifics.

However, we found contrasting results for conspecific seedlings around *S. johorensis* conspecific adult trees. In this case the survival of conspecific seedlings is unaffected by either distance or density. Connell (1971) and Janzen (1970) emphasized that natural enemies must be host specific to generate distance and density dependence to favor heterospecifics. Generalist natural enemies attack wide variety of hosts that could influence the survival of conspecific seedlings at *S. johorensis* adult trees: if the enemies of *S. johorensis* are generalist, then this would weaken density and distance dependence. Another possibility is that density‐dependence may occur at earlier life‐history stages (see below). Recent meta‐analysis by Song et al. (2021) showed that there is large variation of distance—and density‐dependent mortality within genera even if the species are from same family.

### 4.2 Effect of distance and density on growth

We observed that the negative effect of density on height increment in both *P. malaanonan* seedlings and *S. johorensis* seedlings was stronger in high‐density treatments compared to low densities. A study by Linkevičius et al. (2014) demonstrated that intense competition can lead to negative effects on height increment in high‐density treatment plots. Such results suggest that when seedlings occur at high densities, intraspecific resource competition and microbe‐mediated processes in soils can affect the growth of the seedling, resulting in negative density‐dependent processes.

We found that diameter increments of *S. johorensis* seedlings are highly affected by distance and density. Stoll and Newbery (2005) found that conspecifics seedlings and small trees show decreased growth close to adult conspecific trees in dipterocarps. It is possible that adult trees may take phosphorus from conspecific seedlings that occur near to them via the root system. Several studies have suggested that ectomycorrhizal fungi found in the root system would increase phosphorus uptake from nearby nutrient sources and transfer to their host plants (Brearley, 2012; Perez‐Moreno & Read, 2000; Tibbett & Sanders, 2002).

### 4.3 Effect of distance and density on herbivory

Results from our experiment revealed that there is effect of distance and density on herbivory rate in conspecific seedlings. Distance‐dependent herbivory rates on conspecific seedlings were observed. However, no evidence of density dependence was found as herbivory rates in low‐density plots are higher than high‐density plots. It is possible because leaf herbivores are satiated with high densities of seedlings (Aide, 1992; Crawley & Long, 1995).

### 4.4 Lack of density dependent mortality

In this study, we failed to detect density dependence in either species at seedling stage. Several factors may have contributed to these results. First, it could be that the density range used in this study could have been too limited, with only 12 seedlings in the high‐density 1 m^2^ plots. Impacts of density could be more likely to be detected with a larger range of density manipulations. For instance, Watkinson and Harper (1978) demonstrated that negatively density‐dependent relationship was observed in density of greater than 100 flowering plants per 0.25 m^2^. Secondly, density dependence may not have been detected in seedlings of the age that we used in this study. Different life stages such as seed–seedling transition and young seedlings could perhaps experience stronger density dependence because they may be more vulnerable. Several studies found that strong density‐dependent effects in very young seedlings and seed–seedling transitions in tropical species (Freckleton & Lewis, 2006; Silva Matos et al., 1999).

Cannon et al. (2020) similarly reported weak evidence for density‐dependence in a suite of Bornean rainforest trees. Webb and Peart (1999) found evidence for local density‐dependence in the survival of only five of 15 species they tested. Both of these studies found limited evidence of an impact of fungal pathogens in generating mortality or density‐dependence, in contrast with many studies from the neotropics that have found such effects.

### 4.5 Further directions

The Janzen–Connell hypothesis suggests that natural enemies such as insect herbivores and pathogens must be host specific. Host specificity is required to drive Janzen–Connell mechanism in plant communities (Ali & Agrawal, 2012; Clark & Clark, 1984). Dyer et al. (2007) demonstrated that insect herbivores are more specialized in the tropics. However, recent studies found that tropical insect herbivores are more general in their host preferences (Gilbert & Webb, 2007; Novotny & Basset, 2005; Weiblen et al., 2006). In our study, we did not test for the host specificity of natural enemies which is an important element of the Janzen–Connell mechanism. This could highlight the role of host specificity in maintaining forest diversity. Ghazoul (2016) highlighted that how pathogens may be more critical than insects to maintain distance and density dependence in dipterocarp forests. A recent study by Spear and Broders (2021) showed that generalist pathogens contribute to maintenance of forest diversity in tropical area. Thus, experimental studies on density and distance dependence involving natural enemies host specificity on community level could highlight to what extent that natural enemies can maintain forest diversity.

## 5 CONCLUSION

Overall, distance and density‐dependent effects vary for both species tested in this study. Future studies could consider early life history stages (i.e., seed stage, seed–seedling transition, and young seedlings) and whole‐life cycle studies to detect distance and density dependence and their role in maintaining tropical forest diversity. With greater exposure to natural enemies, impacts of distance and density‐dependent effects are more likely to increase.

## AUTHOR CONTRIBUTIONS

**Nazrin Malik:** Conceptualization (equal); data curation (lead); formal analysis (lead); funding acquisition (lead); investigation (lead); methodology (lead); project administration (lead); visualization (equal); writing – original draft (lead); writing – review and editing (equal). **Rob Freckleton:** Conceptualization (supporting); data curation (supporting); formal analysis (supporting); investigation (supporting); methodology (supporting); project administration (supporting); supervision (equal); validation (equal); writing – review and editing (equal). **David P Edwards:** Conceptualization (supporting); investigation (supporting); project administration (supporting); supervision (supporting); visualization (supporting); writing – review and editing (supporting).

## FUNDING INFORMATION

None.

## Supporting information


Figure S1

## Data Availability

All data that support the findings of this study such as experimental datasets, analyses and supporting figures are available on Data Dryad at https://doi.org/10.5061/dryad.9cnp5hqp9.
