# Supplementary material for: Distance and density dependence in two native Bornean dipterocarp species
Source: Ecol Evol. 2023 Apr 19;13(4):e10004. doi: 10.1002/ece3.10004 (PMC10115900; doi:10.1002/ece3.10004)
Supplement: Supplementary file 1 — Figure S1 [file ECE3-13-e10004-s001.docx]

**
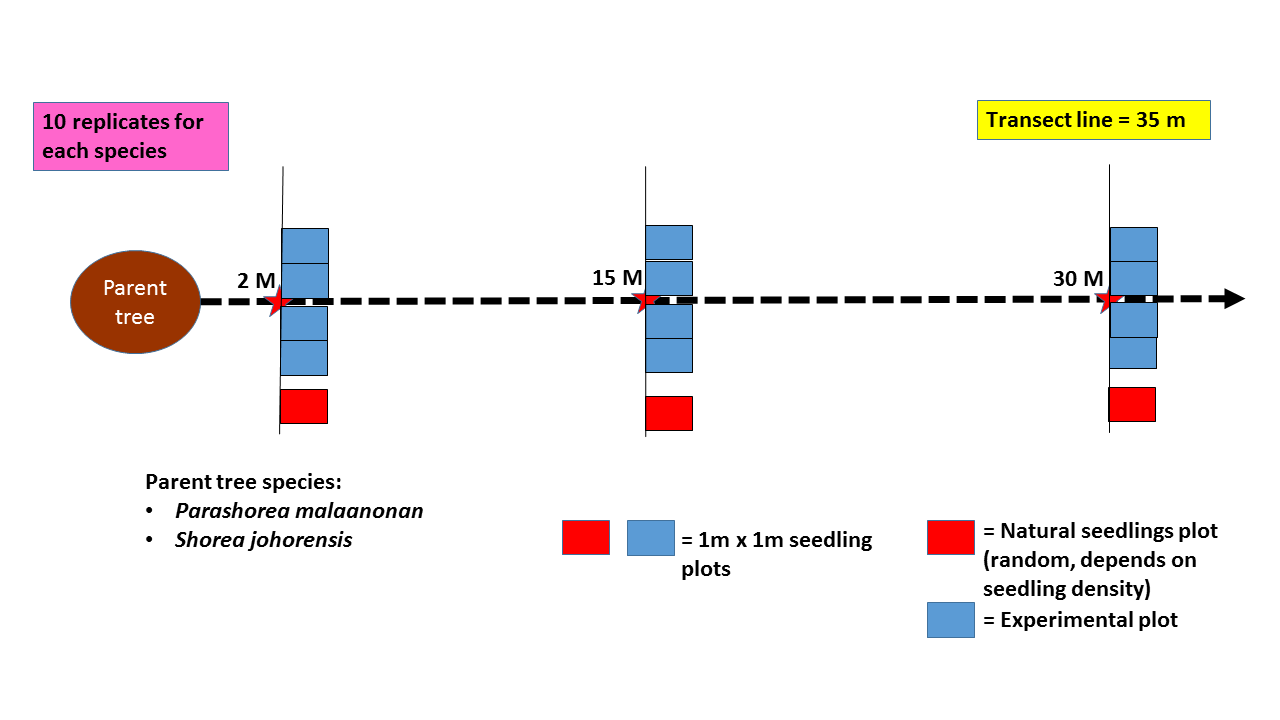
**

**Figure S1**: Study design of distance and density dependence experimental chapter


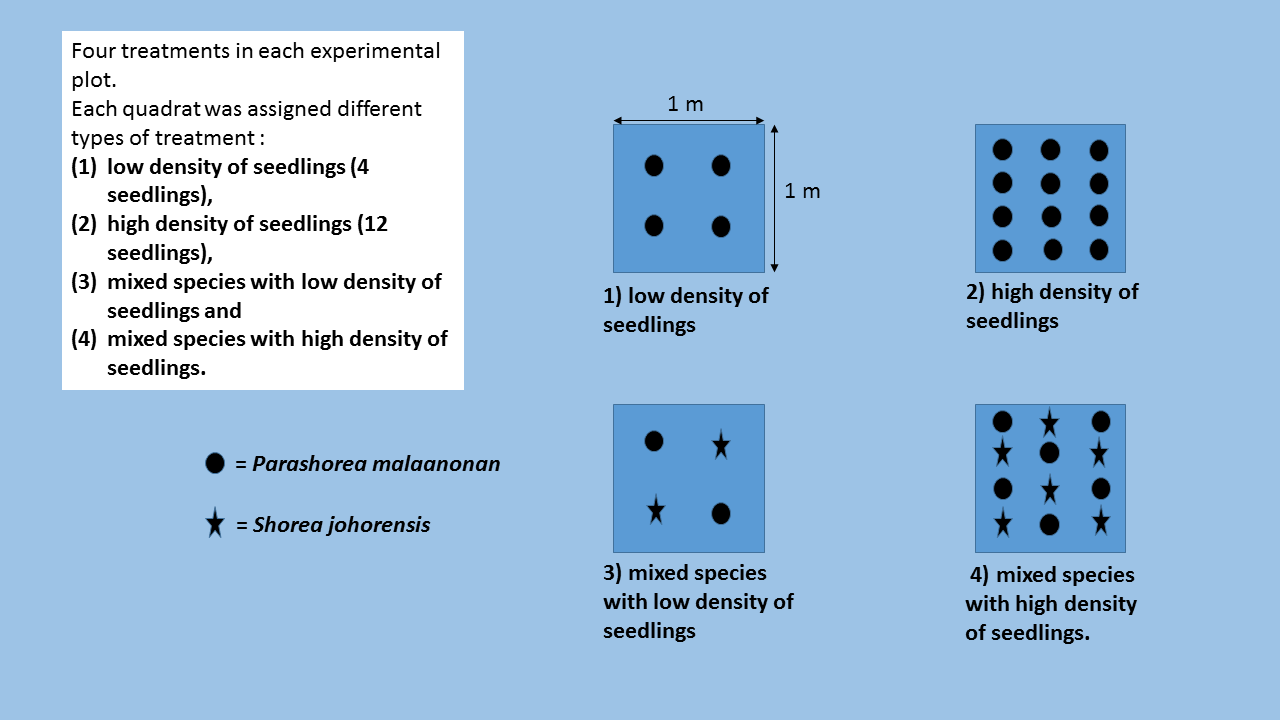


**Figure S2**: Planting treatment applied in the distance and density dependence experimental chapter


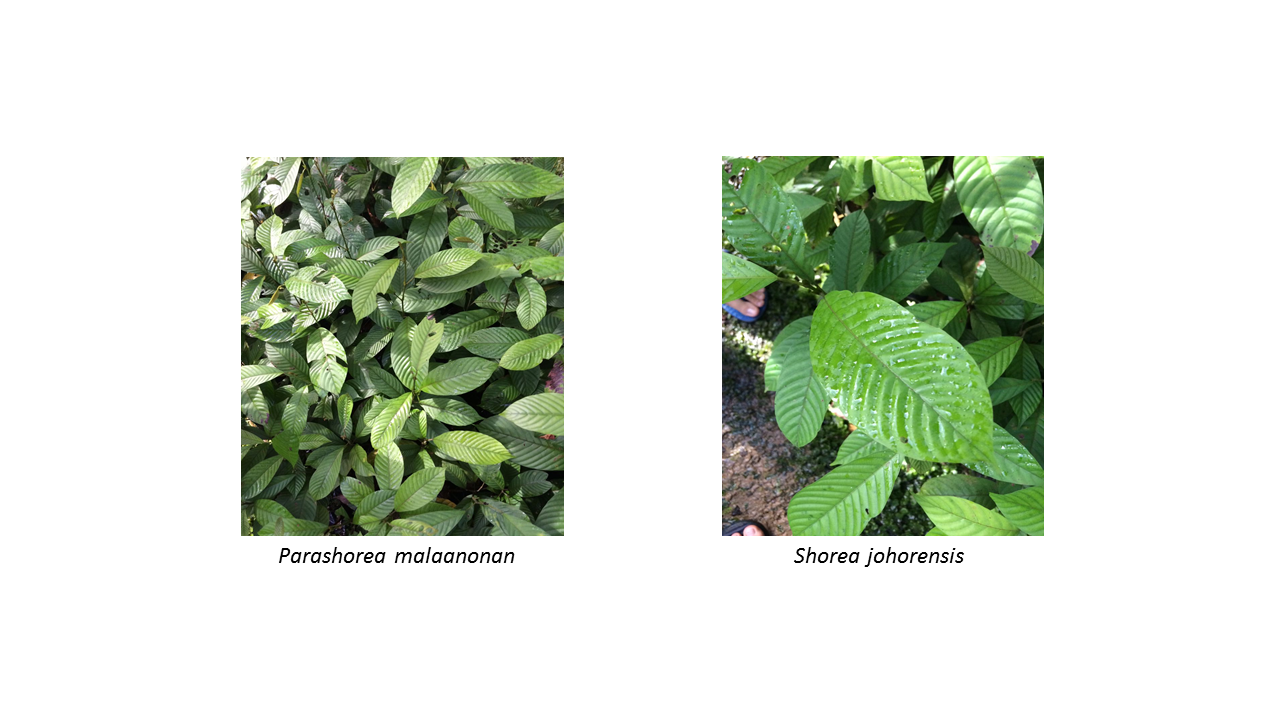


**Figure S3**: Selected dipterocarp species in the distance and density dependence experimental chapter
